# Developing quality indicators for assessing quality of birth centre care: a mixed- methods study

**DOI:** 10.1186/s12884-017-1439-9

**Published:** 2017-08-02

**Authors:** Inge C. Boesveld, Marieke A. A. Hermus, Hanneke J. de Graaf, Marit Hitzert, Karin M. van der Pal-de Bruin, Raymond G. de Vries, Arie Franx, Therese A. Wiegers

**Affiliations:** 1 Jan van Es Institute (Netherlands Expert Centre Integrated Primary Care), Wisselweg 33, 1314 CB Almere, Almere, Netherlands; 2 Department of Child Health, TNO, PO Box 2215 2301, CE Leiden, Leiden, Netherlands; 3 Department of Obstetrics and Gynaecology, Erasmus University Medical Centre, PO Box 2014 3000, CA Rotterdam, Rotterdam, Netherlands; 4 Academie Verloskunde Maastricht/Zuyd University, CAPHRI School for Public Health and Primary Care, PO Box 616 6200, MD Maastricht, Maastricht, Netherlands; 5 Division Woman and Baby, University Medical Centre Utrecht, PO Box 85500 3508, GA Utrecht, Utrecht, Netherlands; 6 NIVEL (Netherlands Institute for Health Services Research), PO Box 1568 3500, Utrecht, BN Netherlands

**Keywords:** Quality indicators, Birthing centres, Structure and process assessment, Delphi method, The Netherlands

## Abstract

**Background:**

Birth centres are described as settings where women with uncomplicated pregnancies can give birth in a home-like environment assisted by midwives and maternity care assistants. If complications arise or threaten, the woman is referred to a maternity unit of a hospital where an obstetrician will take over responsibility. In the last decade, a number of new birth centres have been established in the Netherlands, based on the assumption that birth centres provide better quality of care since they offer a better opportunity for more integrated care than the existing system with independent primary and secondary care providers. At present, there is no evidence for this assumption. The Dutch Birth Centre Study is designed to present evidence-based recommendations for organization and functioning of future birth centres in the Netherlands. A necessary first step in this evaluation is the development of indicators for measuring the quality of the care delivered in birth centres in the Netherlands. The aim of this study is to identify a comprehensive set of structure and process indicators to assess quality of birth centre care.

**Methods:**

We used mixed methods to develop a set of structure and process quality indicators for evaluating birth centre care. Beginning with a literature review, we developed an exhaustive list of determinants. We then used a Delphi study to narrow this list, calling on experts to rate the determinants for relevance and feasibility. A multidisciplinary expert panel of 63 experts, directly or indirectly involved with birth centre care, was invited to participate.

**Results:**

A panel of 42 experts completed two Delphi rounds rating determinants of the quality of birth centre care based on their relevance (to the setting) and feasibility (of use). A set of 30 determinants for structure and process quality indicators was identified to assess the quality of birth centre care in the Netherlands.

**Conclusions:**

We identified 30 determinants for structure and process quality indicators concerning birth centre care. This set will be validated during the evaluation of birth centres in the Dutch Birth Centre Study.

## Background

Internationally, birth centres are described as settings where women with uncomplicated pregnancies can give birth in a home-like environment. In the Netherlands, women with uncomplicated pregnancies can choose where they want to give birth: at home, in a birth centre or in a hospital [1]. At any location, community midwives are responsible for care during labour and birth as long as it stays uncomplicated. When additional medical assistance is required, the women will receive specialist care under responsibility of an obstetrician at an obstetric unit. Birth care in a birth centre is provided by community midwives, assisted by maternity care assistants. The community midwife accompanies the woman to the centre when labour has started. A maternity care assistant assists the midwife during labour and birth and provides postnatal care to the woman and new-born. Most birth centres do not have a permanent staff of midwives and maternity care assistants. They are only present at the centre when accompanying a woman in labour.

Birth centres have been present in the Netherlands since the nineteenth century [2], but not until the year 2000 did the number of these centres begin to grow considerably. This appeared to be a reaction to a severe shortage of maternity care providers, especially primary care midwives and maternity care assistants but also obstetric nurses in hospitals. Birth centres were seen as a solution, because they reduce the pressure on hospital maternity wards by providing women who do not want to give birth at home with a safe and home-like alternative. And because birth centres allow midwives to supervise multiple births simultaneously, they also reduce the pressure on community midwives. These birth centres were typically built right next to, or within, the walls of a hospital. However, most of them disappeared again when the problem of the shortage of maternity care providers was alleviated by a dropping birth rate following the millennium baby boom.

In recent years perceptions about the safety of the maternity care system in the Netherlands began to change. An important cause for this was the publication of the Euro-Peristat data, alarming the Netherlands because of its relatively high perinatal mortality compared to other European countries [3]. It was suggested that this might be related to the strict division between primary and secondary care in the Dutch maternity care system [4–7]. The basic feature of this system is that for healthy women community midwives or general practioners are the responsible care providers (primary care), and for women with pre-existing and emerging pathology obstetricians are the responsible care providers (secondary care) [8]. Media attention given to the Euro-Peristat data and the report from a special committee set up by the Minister of Health (Steering Group “Pregnancy and Childbirth”) [9] may have attributed to a change in the attitudes and behaviour of Dutch women and their care providers with an increasing number of women choosing, or being referred to, a hospital to give birth [10]: in 2000 30.3% of all births took place at home but this fell to 13.1% in 2015 [11]. More and more healthy women are opting for a hospital birth because they do not feel safe at home, or are asking for referral to receive treatment (i.e. pain medication) that cannot be provided in primary care [12]. Birth centres can be seen as an opportunity to keep these healthy women away from the clinical setting, to provide a safe and home-like alternative, but to be close enough to a hospital to be able to take them in quickly when referral is warranted. In their report, the Steering Group recommended more integration in maternity care, by improved cooperation between primary and secondary care and the introduction of birth centres with close links to hospitals. They also recommended further research on the added value of birth centres [8]. In recent years, following these recommendations, a number of new birth centres have been established in the Netherlands, based on the assumption that birth centres provide better quality of care – as measured by perinatal and maternal outcomes – since they offer a better opportunity for more integrated care than the existing system with independent primary and secondary care providers [13]. At present, there is no evidence for this assumption because there is no reliable way to measure degree and quality of integration in care provision. The Dutch Birth Centre Study is designed to present evidence-based recommendations for organization and functioning of future birth centres in the Netherlands, based on careful assessment of existing birth centres [14]. A necessary first step in this process is development of indicators for measuring the quality of the care delivered in birth centres in the Netherlands.

Although formulated in 1990, the definition of quality of care provided by the Institute of Medicine (IOM) is still widely accepted: “*quality of care is the degree to which health services for individuals and populations increase the likelihood of desired health outcomes and are consistent with current professional knowledge*” [15]. Usually three dimensions of quality of care are distinguished: structure (the capacity to provide high quality care), process, and outcome [16]. Measures of these three dimensions are called indicators. To assess quality of care, indicators should be developed for the seven domains of quality identified by the IOM: effectiveness, safety, timeliness, efficiency, equity, accessibility and patient-centeredness [17]. Internationally, standards for birth centres are available and can provide a tool for measuring the quality of service provided to childbearing families in birth centres [18, 19], but these standards must be adjusted for specific settings of these centres, in our case, the unique maternity care system in the Netherlands. A number of outcome quality indicators are available to assess birth centre care (i.e. perinatal and maternal mortality and morbidity) [20–27], but structure and process indicators, specifically developed for birth centre care, are scarce.

In this article we describe a set of determinants for structure and process indicators for assessing the quality of birth centre care and we explain the approach we used to develop this set. We only describe the development of determinants for structure and process indicators, because a newly validated Optimality Index (OI-NL2015) and a Composite Adverse Outcome Score (CAOS) were used to evaluate outcomes of birth centre care [3].

## Methods

### Study design

In order to develop a comprehensive set of structure and process quality indicators to evaluate birth centre care, we used mixed methods. Three phases were followed in the development process: 1) identification of existing quality indicators in birth care, 2) translating these structure and process indicators into determinants, 3) determinant selection by Delphi consultation. The study was conducted in the first half of 2013 as part of the Dutch Birth Centre Study [3].

1. Identification of existing quality indicators in birth care

In the first phase of the study, we used various sources to find existing quality indicators in birth care. We began with an Internet search for documents from Dutch Institutes that had developed quality indicators for maternity care. Documents that described the (development of) quality indicators by midwives, obstetricians and maternity care assistants were obtained. Next, we reviewed international scientific literature about birth centres in order to identify existing quality indicators. We searched PubMed and the Cochrane Library using the Mesh terms: “birthing centres”, “quality indicator”, “health care” and search terms “quality” and “birth centre”. We used references from these articles to find other relevant articles and documents related to quality indicators in maternity care.

2. Translating indicators into determinants

In the second phase we translated the structure and process indicators that we had identified into determinants (or topics): elements that identify the nature of the indicator. We used a framework based on the seven domains of quality according to the IOM (effectiveness, safety, timeliness, efficiency, equity, accessibility and patient-centeredness.) We added an eighth domain, “Law on the Accessibility of Healthcare Facilities”, because of obligations placed on healthcare facilities by this law in the Netherlands. The research group used their experience to add topics that were missing in the resulting list. No outcome indicators were included. We then created a questionnaire that members of an expert panel could complete in a minimum amount of time in order to maximize our response rate.

3. Determinant selection by Delphi consultation

We initiated an online Delphi study with the goal of obtaining consensus among a group of experts. The online Delphi technique is an anonymously structured approach, in which information is gathered from a group of participants through a number of Delphi rounds. The web-based anonymous nature of the Delphi technique ensures that a single individual cannot dominate the consensus formation. Moreover all participants are equally able to change their opinion in the course of the process [28, 29]. Our Delphi study consisted of two online questionnaires.

### Participants

We selected participants for the expert panel from the Research Advisory Group of the Dutch Birth Centre Study [3], participants of former panels of developing indicators for maternity care in the Netherlands, professionals from different disciplines who are working with or in a birth centre with several years of experience, representatives of health insurance companies, policymakers, clients and advisors in birth care. Of the care providers, only experts who are actually involved in birth (centre) care were invited and all health care disciplines related to birth (centre) care were represented. We included professionals in our heterogeneous expert panel: (11 (community and clinical) midwives, 2 general practitioners, 5 maternity care assistants, 6 obstetricians, 4 paediatricians, 5 obstetrics and gynaecology nurse specialists, 7 managers from birth centres, 5 representatives from health insurance companies, 3 representatives from clients and 15 other experts (i.e. policymakers, advisors and research experts). We limited the number of participating clients, because their view on quality of birth centre care is examined in another part of the study [3].

#### Rating determinants by experts: First Delphi round

In May 2013, we sent a link to an online questionnaire by e-mail to the expert panel. The experts were instructed to rate the determinants on relevance (to the setting) and feasibility (of use) and, if necessary, to comment on them. Each determinant was rated on a seven-point Likert scale (1 = not at all relevant/feasible; 4 = neutral; 7 = very much relevant/feasible). Finally, experts were encouraged to suggest additional relevant subjects that should be taken into consideration in the assessment of the quality of birth centre care. All ratings from the first Delphi round were analysed in Excel and distributions of scores were presented in median scores for each determinant. We considered determinants with a median score of ≥6 with agreement to be relevant and feasible to collect and accepted these immediately. Agreement was defined when 80% or more of the ratings were within a range of three (i.e. 5–6-7 of 4–5-6). Determinants that scored with a median score of ≤2 were rejected. Median scores of >3 and <6 with agreement or ≥6 without agreement were discussed again in the second Delphi round. Furthermore, all the comments on determinants from the first round were analysed and the descriptions of determinants were re-phrased in cases of ambiguity. All proposed new determinants from the first round were categorized in domains. New determinants were coded and two researchers of our research group decided, using a consensus method, which determinants should be submitted in the second round. Items the research group already had decided to include in the overall study (i.e. professional experiences and topics related to integration) were not included in the second round.

#### Rating determinants by experts: Second Delphi round

In the second Delphi round, the experts were informed about the median scores on relevance and feasibility of the total expert group, their own scores and the comments of the respondents regarding determinants for which no consensus was reached in the first round. They were instructed to re-consider their rating of the determinants presented in the first round as well as to rate and comment on the new elements the same way as in the first round. This was done to allow experts to revise their opinion of the first round while considering the ratings and comments provided by the other members of the expert panel. The link to the personalized online questionnaire was sent by email 10 days after the first round. Again, the median scores and the degree of agreement were calculated. Only scores ≥6 with agreement were adopted into the list. Determinants with scores for relevance ≥6 with agreement, but feasibility between 3 and 6 were presented to the research group for a final decision.

## Results

**Fig. 1:**
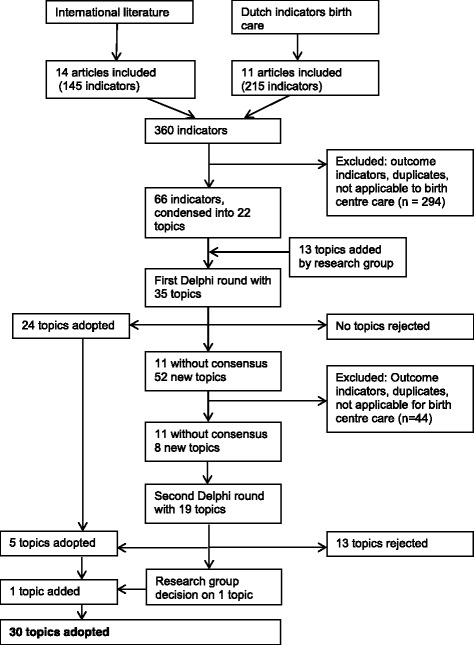
Flowchart selection process indicators quality birth centres

Figure 1 shows the total process that led to the selection of structure and process quality indicators of birth centres, and the number of determinants (topics) at each step.

### Identification of existing quality indicators in birth care

Two hundred fifteen indicators were derived from Dutch sources, 145 from international literature. We eliminated duplication and excluded all outcome indicators. Indicators that clearly do not determine quality of care in a birth centre (because it is clear that this kind of care does not occur in birth centres, i.e. caesarean section) were also excluded from this list. Finally 66 structure and process indicators were identified.

### Translating indicators into determinants

These 66 literature-based indicators were divided into seven themes matching the seven domains of quality according to the United States Institute of Medicine (IOM). The research group added a domain “Law Accessibility of Healthcare Facilities”. In these eight domains, 22 topics were identified, because several indicators appeared to relate to the same topic, albeit with different wordings. The research group added another 13 topics that they missed, based on their experience. After this process, the topics were formulated as 35 determinants to be included in the first questionnaire for the online Delphi panel.

### Determinant selection by Delphi consultation

The questionnaire in the first Delphi round was completed by 48 experts (response rate of 76%). 42 of them also completed the questionnaire in the second round (response rate of 88%). During the first round, 24 of the 35 determinants were accepted for inclusion, none were rejected right away, leaving 11 topics without consensus. 22 experts mentioned 52 new topics they missed in the questionnaire. These topics were labelled and categorized, after which two researchers of our research group decided, based on consensus, that 8 of them would be included in the second Delphi round. In the second round, the 11 topics from the first round on which no consensus was reached and the 8 new topics were presented to the expert panel. This resulted in the acceptance of another five determinants and the rejection of 13 determinants. One determinant was presented to the research group because of low feasibility according to the experts. The research group accepted this determinant, so finally 30 determinants resulted from the Delphi consultation. Table 1 shows the selected determinants per IOM quality domain. Table 2 shows all determinants included in the Delphi procedure with the number or rated scores on the Likert Scale.

**Table 1:** Selected determinants per domain

Determinant	Type of indicator	Rating on:	Median score Likert scale	Consensus (%)	Conclusion round 1	Conclusion round 2
			(1–7)		(80% consensus)	(80% consensus)
Domain: effectiveness						
Written agreements on care aspects (i.e. by hospital care, obstetricians)	Structure	Relevance	7	100	Include	
		Feasible	6	87,5		
Structural evaluation of the care provided in the birth centre	Structure	Relevance	7	93,7	Include	
		Feasible	6	83,4		
Maternity care assistant present during labour^a^	Process	Relevance	6	87,5	Include	
		Feasible	6,5	79,2		
(Integrated) ICT system with hospital and midwifery practices	Structure	Relevance round 1	6	75	Submit again	
		Feasible round 1	5	54,3		
		Relevance round 2	6	90,5		Include
		Feasible round 2	6	76,2		
Domain: safety						
Facilities at a birth centre in relation to emergency care (i.e. CPR resuscitation)	Structure	Relevance	7	95,9	Include	
		Feasible	7	97,9		
Joint (interdisciplinary) emergency care training	Process	Relevance	6	95,8	Include	
		Feasible	6	87,5		
Agreements with ambulance service and nearest hospital about urgent referrals	Structure	Relevance	7	89,6	Include	
		Feasible	6	77		
Domain: timeliness						
Necessary transport time from birth centre to hospital	Process	Relevance	7	100	Include	
		Feasible	7	96		
In case of referral from the birth centre durante partu: required time between decision to refer and treatment in hospital	Process	Relevance	7	95,9	Include	
		Feasible	6	81,3		
Domain: efficiency						
In case of referral from the birth centre durante partu: guaranteed access to the hospital with which agreements were made	Process	Relevance	7	100	Include	
		Feasible	6	87,6		
Distance between birth centre and hospital	Structure	Relevance	7	98	Include	
		Feasible	7	96		
Cooperation with (almost) all relevant organizations in the region (such as midwifery practices and maternity care assistance organisations)	Process	Relevance	6	89,5	Include	
		Feasible	6	81,3		
Protocols on care aspects	Structure	Relevance	7	87,5	Include	
		Feasible	6,5	81,3		
Participation of birth centre in local maternity care consultation and cooperation group (VSV)	Process	Relevance	7	85,4	Include	
		Feasible	6	81,3		
Indoor connection between birth centre and hospital	Structure	Relevance	6	84	Include	
		Feasible	7	96		
Joint use of an electronic patient record	Structure	Relevance round 1	6	87,6	Submit again	
		Feasible round 1	6	66,7		
		Relevance round 2	6	95,2		Include
		Feasible round 2	6	85,7		
System of quality improvement (i.e. accreditation)	Structure	Relevance round 1	6	70,9	Submit again	
		Feasible round 1	5	56,3		
		Relevance round 2	6	85,7		Decision Research group: include
		Feasible round 2	5	80,9		
Multidisciplinary education as result of formulated points of improvement from perinatal audit	Process	Relevance round 2	6	90,5	New in round 2	Include
		Feasible round 2	6	83,3		
Domain: equity						
Care pathways formulated with chain partners	Structure	Relevance	6	95,9	Include	
		Feasible	6	79,2		
Birth centre has vision of birth care	Structure	Relevance	7	91,8	Include	
		Feasible	6	75		
Formal partnership agreement with chain partners	Structure	Relevance	6	83,4	Include	
		Feasible	7	81,3		
Admission agreement for professionals who use birth care facilities at the birth centre	Structure	Relevance round 1	6	69,3	Submit again	
		Feasible round 1	7	75,5		
		Relevance round 2	6	81		Include
		Feasible round 2	7	85,7		
Domain: accessibility						
24 /7 telephone accessibility birth centre	Process	Relevance	7	100	Include	
		Feasible	7	98		
Physical access to birthing centre for clients (i.e. parking)	Structure	Relevance	7	96	Include	
		Feasible	6	78		
Physical access to birthing centre for midwives and maternity care assistants (e.g. parking)	Structure	Relevance	6	92	Include	
		Feasible	6	80		
Domain: patient-centeredness						
Facilities at a birth centre in relation to pain management (i.e. nitrous oxide)	Structure	Relevance	6	100	Include	
		Feasible	6	83,7		
Continuous presence of a healthcare provider during labour^a^	Process	Relevance	7	98	Include	
		Feasible	6	81,3		
Structural research on client experiences	Structure	Relevance	7	98	Include	
		Feasible	6	85,5		
Focusing on the patients (i.e. use individual birth plan)	Process	Relevance	6	89,6	Include	
		Feasible	6	83,4		
Participation and representation of clients in organisation (i.e. in the board)	Structure	Relevance round 2	6	85,7	New in round 2	Include
		Feasible round 2	6	78,6		

^a^These determinants appear similar but are different: ‘Continuous presence of a healthcare provider during labour’ refers to continuous support of labour (not leaving alone the woman in labour). ‘Maternity care assistant present during labour’ refers to the presence of assistance of the midwife during childbirth. In the Netherlands, the midwife attends birth of low risk women, regardless the location (at home, in a birth centre or in a hospital) and is assisted by a maternity care assistant. Sometimes, it happens that the maternity care assistant is too late present at the childbirth to assist the midwife adequately. This determinant refers to this aspect

**Table 2:** All determinants included in the Delphi-procedure

			Scores Likert Scale (N)												
Determinant	Domain^a^	Rating on:	1	2	3	4	5	6	7	Do not know	Total (N)	Median	Consensus (%)	Conclusion round 1 (80% consensus)	Conclusion round 2 (80% consensus)
Adopted after first round															
Necessary transport time from birth centre to hospital	3	Relevance					3	13	34		50	7	100	Include	
		Feasible				2	4	14	30		50	7	96		
24 /7 telephone accessibility birth centre	6	Relevance			2			6	42		50	7	100	Include	
		Feasible			1	3		10	36		50	7	98		
Facilities at a birth centre in relation to pain management (i.e. nitrous oxide)	7	Relevance	1	1	2	2	12	17	14		49	6	100	Include	
		Feasible	1		3	3	5	14	22	1	49	6	83,7		
Written agreements on care aspects (i.e. by hospital care, obstetricians)	1	Relevance					4	17	27		48	7	100	Include	
		Feasible		1	1	2	4	18	20	2	48	6	87,5		
In case of referral from the birth centre durante partu: guaranteed access to the hospital with which agreements were made	4	Relevance					3	9	36		48	7	100	Include	
		Feasible			1	4	9	12	21	1	48	6	87,6		
Distance between birth centre and hospital	4	Relevance				1	7	15	27		50	7	98	Include	
		Feasible				2	1	14	33		50	7	96		
Continuous presence of a healthcare provider during labour	7	Relevance				1	6	14	27		48	7	98	Include	
		Feasible		1	5	3	12	14	13		48	6	81,3		
Structural research on client experiences	7	Relevance				1	2	18	27		48	7	98	Include	
		Feasible	1		2	4	3	15	23		48	6	85,5		
Physical access to birthing centre for clients (i.e. parking)	6	Relevance				2	6	16	26		50	7	96	Include	
		Feasible		1	2	7	2	13	24	1	50	6	78		
Care pathways formulated with chain partners	5	Relevance				1	7	18	21	1	48	6	95,9	Include	
		Feasible		1		8	7	14	17	1	48	6	79,2		
Facilities at a birth centre in relation to emergency care (i.e. CPR resuscitation)	2	Relevance				2	4	11	32		49	7	95,9	Include	
		Feasible				1		10	37	1	49	7	97,9		
In case of referral from the birth centre durante partu: required time between decision to refer and treatment in hospital	3	Relevance				1	3	12	31	1	48	7	95,9	Include	
		Feasible			3	3	9	10	20	3	48	6	81,3		
Joint (interdisciplinary) emergency care training	2	Relevance				2	7	17	22		48	6	95,8	Include	
		Feasible			1	5	7	19	16		48	6	87,5		
Structural evaluation of the care provided in the birth centre	1	Relevance				2	1	16	28	1	48	7	93,7	Include	
		Feasible			2	4	8	18	14	2	48	6	83,4		
Physical access to birthing centre for midwives and maternity care assistants (e.g. parking)	6	Relevance				4	9	17	20		50	6	92	Include	
		Feasible		1	2	6	3	15	22	1	50	6	80		
Birth centre has vision of birth care	5	Relevance		1		3	3	15	26		48	7	91,8	Include	
		Feasible		1	4	7	5	11	20		48	6	75		
Cooperation with (almost) all relevant organizations in the region (such as midwifery practices and maternity care assistance organisations)	4	Relevance	1	1		3	4	16	23		48	6	89,5	Include	
		Feasible		2	1	6	8	14	17		48	6	81,3		
Agreements with ambulance service and nearest hospital about urgent referrals	2	Relevance	2			3	4	9	30		48	7	89,6	Include	
		Feasible	1		1	8	4	10	23	1	48	6	77		
Focusing on the patients (i.e. use of individual birth plan)	7	Relevance				4	2	24	17	1	48	6	89,6	Include	
		Feasible			2	4	14	13	13	2	48	6	83,4		
Maternity care assistant present during labour	1	Relevance			2	4	7	12	23		48	6	87,5	Include	
		Feasible		2	2	5	2	12	24	1	48	6,5	79,2		
Protocols on care aspects	4	Relevance	2		1	2	3	10	29	1	48	7	87,5	Include	
		Feasible	3		1	4	3	12	24	1	48	6,5	81,3		
Participation of birth centre in local maternity care consultation and cooperation group (VSV)	4	Relevance	2			5	1	10	30		48	7	85,4	Include	
		Feasible	1		1	6	3	13	23	1	48	6	81,3		
Indoor connection between birth centre and hospital	4	Relevance	2		1	5	8	11	23		50	6	84	Include	
		Feasible				2	2	13	33		50	7	96		
Formal partnership agreement with chain partners	5	Relevance	2	1		5	5	15	20		48	6	83,4	Include	
		Feasible		1	1	7	3	10	26		48	7	81,3		
Adopted after second round															
Joint use of an electronic patient record	4	Relevance round 1				6	3	19	20		48	6	87,6	Submit again	
		Feasible round 1	1	2	3	8	5	8	19	2	48	6	66,7		
		Relevance round 2	1			1	1	20	19		42	6	95,2		Include
		Feasible round 2	1		2	3	5	16	15		42	6	85,7		
(Integrated) ICT system with hospital and midwifery practices	1	Relevance round 1		1		9	3	17	16	2	48	6	75	Submit again	
		Feasible round 1	1	3	4	12	3	9	14	2	48	5	54,3		
		Relevance round 2				3	6	19	13	1	42	6	90,5		Include
		Feasible round 2	1		2	5	10	13	9	2	42	6	76,2		
System of quality improvement (i.e. accreditation)	4	Relevance round 1			1	10	8	12	14	3	48	6	70,9	Submit again	
		Feasible round 1	2	1	1	7	9	11	11	6	48	5	56,3		
		Relevance round 2	1			4	8	16	12	1	42	6	85,7		decision Research group: include
		Feasible round 2	2		1	3	15	10	9	2	42	5	80,9		
Admission agreement for professionals who use birth care facilities at the birth centre	5	Relevance round 1	1		3	4	6	10	18	7	49	6	69,3	Submit again	
		Feasible round 1			1	3	3	8	26	8	49	7	75,5		
		Relevance round 2	1		1	3	4	13	17	3	42	6	81		Include
		Feasible round 2				5		7	29	1	42	7	85,7		
Multidisciplinary education as result of formulated points of improvement from perinatal audit	4	Relevance round 2	1			3	1	17	20		42	6	90,5		Include
		Feasible round 2			1	5	3	16	16	1	42	6	83,3		
Participation and representation of clients in organisation (i.e. in the board)	7	Relevance round 2	1			5	12	14	10		42	6	85,7		Include
		Feasible round 2	1			7	7	9	17	1	42	6	78,6		
Rejected after round 2															
Opportunities to stay in the birth centre after giving birth	7	Relevance round 1	2	2	4	15	9	11	6		49	5	71,4	Submit again	
		Feasible round 1	1	1	3	5	6	13	19	1	49	6	77,5		
		Relevance round 2	2	4	5	7	10	9	5		42	5	61,9		Reject
		Feasible round 2			3	4	3	14	18		42	6	83,3		
Legal entity (i.e. foundation, association)	8	Relevance round 1	2	2	5	16	7	8	4	5	49	4	57,2	Submit again	
		Feasible round 1		1	1	8	2	10	21	6	49	6	67,4		
		Relevance round 2	3	3	1	20	3	6	2	4	42	4	57,1		Reject
		Feasible round 2				5	4	11	19	3	42	6	80.9		
Independent supervisory board	8	Relevance round 1	2	2	2	7	6	13	13	3	48	6	66,7	Submit again	
		Feasible round 1	2	2		7	3	12	20	2	48	6	73		
		Relevance round 2		1	1	6	5	17	11	1	42	6	78,6		Reject
		Feasible round 2			1	7	2	17	14	1	42	6	78,6		
Multidisciplinary composition of the board of the birth centre (e.g. midwives, maternity care organization and hospital)	4	Relevance round 1	3	1	2	4	10	11	17		48	6	79,1	Submit again	
		Feasible round 1	1		1	5	5	8	28		48	7	85,4		
		Relevance round 2	1	1	1	6	5	18	10		42	6	78,6		Reject
		Feasible round 2				3	2	9	28		42	7	92,7		
Publication of annual report	8	Relevance round 1	2		1	8	9	14	14		48	6	77,2	Submit again	
		Feasible round 1		2		4	2	14	25	2	49	7	85,5		
		Relevance round 2		1		8	7	14	12		42	6	78,6		Reject
		Feasible round 2				6		7	28	1	42	7	83,4		
Number of births and postpartum stays that occur per year in the birth centre	4	Relevance	2	2	2	6	7	13	16		48	6	75	submit again	
		Feasible			1	2	5	15	25		48	7	93,8		
		Relevance round 2	2	1		6	5	21	7		42	6	78,6		Reject
		Feasible round 2				1		8	33		42	7	97,6		
Facilities at a birth centre in relation to stay (i.e. possibilities for father to stay overnight, private shower and/or toilet in room, bath)	7	Relevance round 1		2	4	4	10	13	16		49	6	79,6	Submit again	
		Feasible round 1			1	6	6	10	26		49	7	85,7		
		Relevance round 2		1	4	4	8	12	13		42	6	78,7		Reject
		Feasible round 2			1	1	4	8	27	1	42	7	92,8		
24 h presence of a caregiver in birth centre (maternity care assistant or midwife)	2	Relevance round 2	3	3	2	4	4	13	12	1	42	6	69.1		Reject
		Feasible round 2	1	2	1	2	2	8	24	2	42	7	80,9		
Independent or freestanding setting with a home-like, non-clinical atmosphere	6	Relevance round 2	4	1	8	8	7	9	5		42	4,5	54,7		Reject
		Feasible round 2		2	2	5	6	14	13		42	6	78,6		
Detailed business plan for the birth centre	4	Relevance round 2		2	1	8	7	15	6	3	42	5,5	71,4		Reject
		Feasible round 2			1	4	4	15	17	1	42	6	85,7		
Participation of the birth centre in scientific research	1	Relevance round 2	1		3	10	14	6	7	1	42	5	71,4		Reject
		Feasible round 2	1			7	8	12	13	1	42	6	78,6		
Transfer from birthing room to a residence room when the mother of newly-born child wants/can/ has to stay a (part of her) childbed period;	7	Relevance round 2	2	3	1	14	6	8	2	6	42	4	50		Reject
		Feasible round 2	1	1	1	7	5	9	12	6	42	5,5	50		
Staff in the birth centre mirrors the population that (may) use the birth centre	7	Relevance round 2	9	3	4	13	4	3	2	4	42	4	50		Reject
		Feasible round 2	4	4	1	12	3	10	5	3	42	4	38,1		

^a^Description of Domains:

1: Effectiveness

2: Safety

3: Timeliness

4: Efficiency

5: Equity

6: Accessibility

7: Patient-centeredness

8: Law on accessibility of health care facilities

## Discussion

In this study, part of the Dutch Birth Centre Study, we identified a set of 30 determinants, to be translated into 30 structure and process quality indicators that can be used to assess the quality of birth centre care in the Netherlands. The new developed determinants are derived from existing quality indicators in maternity care in the Netherlands (used to measure quality of care by midwives, obstetricians and maternity care assistants) and indicators derived from international documents concerning birth centre care. The experts selected 5 determinants that are used by Laws in the research on characteristics and practices of birth centres in Australia [20] and 4 determinants derived from Dutch existing quality indicators. They also selected 3 determinants which were formulated in a quality framework of birth centre care, proposed by the Royal Dutch Organization of Midwives (KNOV). Ten selected determinants are used by different organizations to assess quality of care (e.g. maternity care assistance, emergency care) [30–34]. Finally, 7 new determinants were selected by the experts. The final set of indicators will be included in the on-going study to evaluate birth centre care in the Netherlands.

### Strength

A strength of the development of this set of determinants for indicators is that it is developed in collaboration with all parties involved in birth centre care, and is based on consensus. Therefore it can be expected that all professionals in the field will accept assessing the quality of birth centre care using this set of indicators.

### Limitations

We are aware that the set of determinants for indicators we developed has its limitations. Firstly, to assess care in general, structure, process and outcome indicators should be used. However, because there are already a large number of quality indicators to assess outcomes of birth centre care, this set contains only structure and process indicators [35]. The expert panel chose 19 structure and 11 process indicators to asses birth centre care. For the same reason the set we developed does not include indicators of women’s experiences of care, because they can be regarded as outcome indicators [36]. Thirdly, this set only consists of determinants for indicators. The process for developing structure and process quality indicators for birth centres still needs to be described. Also, we do not yet know whether this set of determinants for indicators will be able to differentiate between birth centres or not. It has yet to prove itself in practice: the Dutch National Birth Centre Study will be the first to use these indicators to assess the quality of care.

Finally, although our study was focused on Dutch birth centres, we expect that this set of determinants for indicators will be applicable in other settings where birth centres are used.

## Conclusions

We used an online Delphi-method to develop a list of thirty determinants for structure and process indicators to measure quality of birth centre care. We will describe the process for developing quality indicators from these determinants and evaluation of the validity and reliability of these indicators as part of the Dutch Birth Centre Study in a later paper. It is important to underscore that indicators are part of an on-going cycle of quality improvement. Indicators should never be static. Changes in evidence or clinical relevance, a consistently high performance or a low variation in achievement, new developments and demographic changes in the population of childbearing women, all may be criteria for removing an indicator or adding a new one in a future list of determinants for quality indicators for birth centre care.

## Abbreviations

IOM: Institute of Medicine
KNOV: Royal Dutch Organization of Midwives
ZonMw: the Netherlands Organisation for Health Research and Development ZonMw
